# The Role of Information Visualisation and Anecdotal Evidence in Medical Students’ Clinical Reasoning Process: A Cross-Sectional Survey Study

**DOI:** 10.1177/23821205241293491

**Published:** 2024-11-21

**Authors:** Anna Janssen, Micah B. Goldwater, Courtney B. Hilton, Carissa Bonner, Tim Shaw

**Affiliations:** 1 Faculty of Medicine and Health, 4334 The University of Sydney, Sydney, NSW, Australia.; 2 School of Psychology, 4334 The University of Sydney, Sydney, NSW, Australia.; 3 School of Psychology, 1415 University of Auckland, Auckland, New Zealand.; 4 Menzies Centre for Health Policy & Economics, School of Public Health, Faculty of Medicine and Health, University of Sydney, Sydney, NSW, Australia.

**Keywords:** clinical reasoning, medical students, informatics digital health, data visualisation, data literacy

## Abstract

**BACKGROUND:**

Medical students are often taught clinical reasoning implicitly, rather than through a formal curriculum. Like qualified health professionals, they engage in a wide range of information seeking and other practices as part of the clinical reasoning process. This increasingly includes seeking out information online and being informed by anecdotal information from social media or peer groups. The aim of this research was to investigate how anecdotes and icon arrays influenced the clinical reasoning process of medical students deciding to prescribe a hypothetical new drug.

**METHODS:**

A cross-sectional survey design was used. The survey required participants to respond to six hypothetical clinical scenarios in which they were asked to prescribe a hypothetical drug “polypill” for a specific patient. The order of delivery of the six scenarios was randomised for each participant. In response to each scenario, participants indicated how effective they perceived each drug to be. The study received ethics approval from the University of Sydney Human Research Ethics Committee: Protocol No: 2019/001. All participants provided written informed consent before agreeing to participate in the study.

**RESULTS:**

A total of 56 medical students fully completed the survey. Statistical analysis of the responses indicated that the icon array may be effective for highlighting how the polypill reduces CVD risk, reducing the impact of anecdotes on efficacy judgments. Without the icon array, both the positive and negative anecdotes made participants less willing to prescribe the polypill.

**CONCLUSIONS:**

Medical student clinical reasoning processes appear to be influenced by anecdotal information and data visualisations. The extent of this influence is unclear, but there may be a need to actively educate students about the influence of these factors on their decision-making as they graduate into a world where they will be increasingly interacting with anecdotal information on social media and visualisations of electronic data.

## Introduction

Clinical reasoning describes the conscious and unconscious cognitive processes used to understand contextual patient factors, preferences and characteristics and the practice environment to support decision-making and manage patients.^1^ It is considered a core competency for medical practitioners.^1,2^ Despite this it is often taught implicitly, not explicitly to medical students.^3,4^ As the development of clinical reasoning skills is often self-directed for medical students, it is important to understand the factors that influence information seeking and other components of the clinical reasoning process. Increasingly, there is an expectation that medical schools will explicitly teach clinical reasoning to medical students as part of a formal curriculum^3^ and that student competence in this area should be robustly assessed.^1^ One reason for this is that while medical students have strategies to support the clinical reasoning process, they are often unprepared for the complexity of real-world clinical decision-making.^2^

Clinical reasoning involves a range of components including information gathering and seeking, hypothesis generation, problem representation, selecting a working diagnosis and developing a management plan.^1^ Information gathering and seeking is an increasingly crucial skill for medical practitioners who need to find, appraise and synthesise large quantities of information to make clinical decisions.^5^ The skill is central to optimal delivery of patient-centred care informed by the principles of evidence-based medicine (EBM).^5,6^ As such, medical students need to know how to navigate and synthesise vast amounts of information in order to be prepared for practice upon graduation from their degrees.^7^ Medical students make many decisions when they are information seeking and gathering evidence-based information,^8^ and there is evidence that students closer to graduation are more effective at information seeking than those earlier in their degrees.^7^ As is the case with medical practitioners, speed and convenience are important factors when medical students are determining how and where they will source information.^5,8^ However, it is unclear whether there is a relationship between efficient information seeking practices and effective synthesis of information.^7^

Increasingly, online resources are being used by medical students to find both general medical evidence and also clinical, drug and diagnostic information.^7^ Online search engines such as Google and resources such as Wikipedia are known sources of online information for medical students, although it has been noted these are often a first source of information rather than an end point.^5,8^ Social influences also play a role in medical student information behaviours. Teachers with medical degrees are known to be a key source of referrals for online resources.^9^ Information recommended by a mentor or peer is more likely to be used by medical students in clinical decision-making than other information.^8^ Medical students are also known to use social media to maintain personal connections extensively.^8^ Furthermore, one study into medical students’ willingness to receive a COVID-19 vaccine suggests that recommendations of friends and family, particularly parents, have a significant influence on willingness to be vaccinated.^10^ This broadly aligns with research into the general population which suggests that peer and anecdotal information can affect health decisions by influencing an individual's perception of the effectiveness of treatments^11^ and vaccine hesitancy.^12^

In addition to having effective information seeking skills, contemporary medical students also need to have a good level of information literacy due to the large amount of information they will be expected to synthesise when they become medical practitioners.^9^ Today's medical students will be graduating from the highly digitised health sector, in which they will need to obtain and synthesise ever-increasing amounts of information in order to apply evidence-based approaches to care delivery.^5^ They will also be practising in a working environment where information literacy skills and clinical informatics knowledge will be particularly beneficial as information communication technologies, particularly clinical information systems like electronic health records, that generate large amounts of health and biomedical information become increasingly widespread.^13^ Understanding how different ways of presenting information influence the clinical decision reasoning process is a valuable step in improving the delivery of clinical reasoning education in medical curriculums. Visual interfaces, such as dashboards, are an increasingly common tool in healthcare for improving the interpretation and use of health information.^14^ The use of certain types of visual interfaces may also support health decision-making. There is a growing body of research showing icon arrays, a type in which a single shape is repeated a specific number of times and some of the shapes are coloured or altered in some way to show proportion, which may help reduce cognitive bias in patients when making health decisions.^15^ This effect on health decision-making is yet to be explored in the context of medical students.

Furthermore, visualisation may have a role in supporting the synthesis of information and other learning processes. Visualisation of the steps in clinical reasoning has been shown to help medical students externalise the steps involved in complex clinical problem-solving and enhance their approach to information gathering and other aspects of clinical decision-making^16^ and may increase the accuracy of recall of patient case information.^17^ Presenting textual information alongside visualisations has been shown to influence the way learners construct mental models related to that information.^18^ However, the effect is not always beneficial, and when using visualisations to support learning processes, it is important to focus not only on visualising the information but also on ensuring the specific way the information is visualised is appropriate for the task learners are being asked to undertake.

The study described in this manuscript investigates how different mechanisms for presenting information influenced medical students’ clinical reasoning process. To date, limited research has been undertaken into the role of anecdotes, information based on personal experience or observation not collected in a systematic manner, or data visualisation in medical student clinical reasoning. Addressing this gap is important for improving understanding of how the development of clinical reasoning skills could be better developed in medical students, as this may contribute to improving the explicit teaching and assessment of clinical reasoning in medical curriculums.^1,3,19^ Further, we know that all people are influenced by their personal experiences and individual anecdotes, and the effect is stronger for decisions about health than in other areas of life.^20^ This may be why there is evidence that medical practitioners are often more engaged by clinical narratives and individual patient presentations^21^ than by evidence-based guidelines.^22^ There is a clear tension between human nature to be influenced by anecdotes and the principles of evidence-based medicine,^23^ a tension that has been underexplored in the literature. This research is one step towards addressing this gap by exploring how medical students reason when they are presented with both decision supports that visualise evidence and patient anecdotes related to that evidence. Furthermore, the study seeks to understand different mechanisms medical students use to synthesise different types of information as part of clinical decision-making. It is important to explore this as the health sector is increasingly digitised and the amount of information medical students and practitioners need to synthesise to deliver evidence-based medicine is growing at a rapid rate.^5^

## Methods

### Aim/hypothesis

The primary aim of the study was to investigate how anecdotal information and visualisation of adverse outcomes influenced medical students’ clinical reasoning process. In order to investigate this, participants responded to hypothetical scenarios where they were asked to consider whether they would prescribe the fictitious “polypill.” The “polypill” is described as a hypothetical new combination therapy for treating cardiovascular disease.

The study research questions were as follows:

RQ1: What effect would visualisations and anecdotes have on medical students’ perception of the efficacy of a hypothetical new drug for managing the risk of having a cardiovascular disease event (polypill)?

RQ2: What effect would visualisations and anecdotes have on medical students’ willingness to recommend a hypothetical new drug for managing the risk of having a cardiovascular disease event (polypill)?

RQ3: What effect did medical students think showing patients visualisations and anecdotes about cardiovascular disease risk in the consult would have on patient adherence to treatment with a hypothetical new drug for managing the risk of having a cardiovascular disease event (polypill)?

RQ4: What other information do medical students need to consider when engaging in the clinical reasoning process on whether to prescribe a hypothetical new drug for managing the risk of having a cardiovascular disease event (polypill) to patients?

The reporting for this study confirms the consensus-based checklist for reporting of survey studies (CROSS) reporting guidelines.^24^ Refer to Supplementary File 2 for manuscript page numbers for each relevant item for this study.

**Figure 1.**
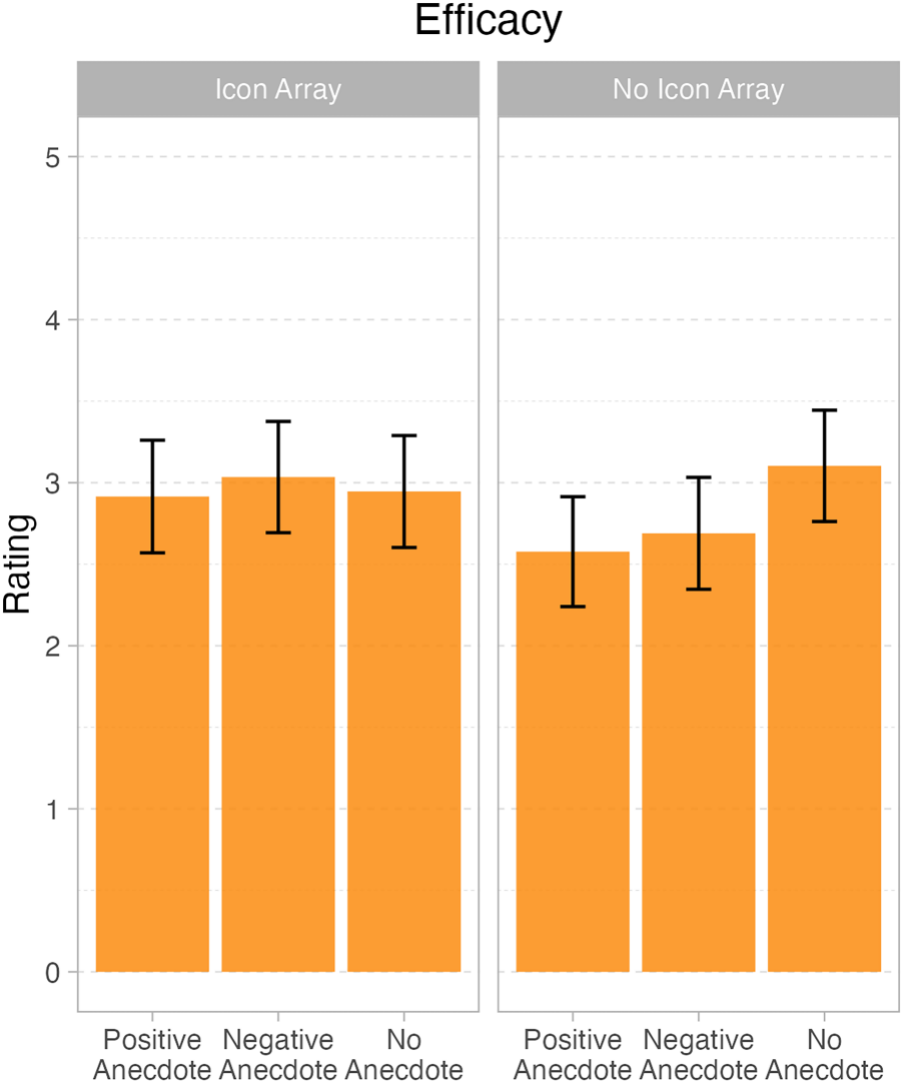
Marginal means and 95% confidence intervals for question 1 – efficacy judgments, displaying the interaction between icon array and anecdote.

### Participants and study setting

Participants were eligible to participate in the study if they were students enrolled in either a postgraduate or undergraduate medical degree at an Australian medical school. Medical degrees in Australia typically focus on theory and classroom-based teaching in the early years, with a transition to clinical placements and other advanced skills training in the later years of the degree. Participant inclusion criteria included being enrolled in a medical degree in Australia at the time of participating in the study. Exclusion criteria included not being enrolled in a medical degree or being enrolled in a medical degree delivered by a tertiary institution outside of Australia.

Participants were recruited via a newsletter or social media notice disseminated by either the medical school or a relevant student association. The newsletter notice provided a link to complete the online survey. Completion of the survey was indicative of consent to participate.

A total of 89 participants attempted the survey, and 56 fully completed all responses. The majority of participants were postgraduate students (91.07%, *n* = 51), with only five enrolled in undergraduate degrees.

### Study design

A cross-sectional survey design was used. The survey required participants to respond to six hypothetical clinical scenarios in which they were asked to prescribe a drug for a specific patient. The order of delivery of the six scenarios was randomised for each participant. The survey platform used a block system to cluster questions and, if randomisation is being used, subsequently assign blocks of questions to each user as they access the survey. In this study, each hypothetical scenario was its own block so the order each case was presented was random for each participant that responded to the survey. In response to each scenario, participants indicated how effective they perceived each drug to be. Refer to Supplementary File 1 for an overview of the hypothetical scenarios.

### Data collection

Data were collected via an anonymous online survey consisting of demographic information and six hypothetical clinical scenarios. The survey was pre-tested by four researchers known to the authors the month before it was distributed to participants. The online survey was distributed between May and December 2021 via a browser-based surveying tool.^25^ Data collection took place between March and December 2021.

Demographic information in the survey included participant age, gender, the Australian state they lived in, whether they were an undergraduate or postgraduate medical student and the year of their medical degree they were currently enrolled in. Participants were also asked how confident they felt prescribing medications in two different scenarios: (1) when there was clear evidence, it would benefit the patient, and (2) when there was conflicting evidence, it would benefit the patient.

The hypothetical clinical scenarios in the survey all consisted of textual information providing background on a randomised controlled trial about the efficacy of a new “polypill” and its side effects. On a five-point Likert scale where 1 indicated “not effective at all” and 5 indicated “extremely effective,” participants were asked to indicate the following: (1) how effective they thought the pill was, (2) how likely they were to recommend the pill to patients and (3) how likely they thought the information presented in the case would be to increase medication adherence if it was shared with the patient. Participants could also provide a free-text response to each case asking if there were any other factors, they might consider in making a decision to prescribe the pill. In addition to this core information, each hypothetical clinical scenario could include a:

- positive anecdote about a patient’s experience with the pill
- negative anecdote about a patient’s experience with the pill
- visualisation (icon array) of the risk of having a CVD event in the next 5 years
- visualisation (icon array) of the risk of having a CVD event in the next 5 years and a negative anecdote about the patient’s experience with the pill
- visualisation (icon array) of the risk of having a CVD event in the next 5 years and a positive anecdote about the patient’s experience with the pill

### Data analysis

Structured data from the survey were analysed by conducting a series of mixed-effects models. These models included categorial fixed factors of icon array (present vs absent), anecdote (none, positive and negative) and their interaction (polypill). The analyses were conducted using R statistical programming language, with lme4 mixed-effects modelling package, and plots generated with ggplot 2.^26–29^

Free-text data from the survey were content analysed by one researcher (AJ). All free-text responses were read through to get a good sense of the data, and key concepts were identified. Relationships between concepts for different hypothetical clinical scenarios were also analysed.

### Ethics

The study received ethics approval from the University of Sydney Human Research Ethics Committee: Protocol No: 2019/001. All participants provided written informed consent before agreeing to participate in the study.

## Results

### Demographics

A total of 89 participants attempted the survey, and 56 fully completed all responses. The majority of participants were postgraduate students (91.07%, *n* = 51), with only five enrolled in undergraduate degrees. We note that all analyses were also conducted with the undergraduate students removed. This made no change to the pattern of results, and so we present analyses below with the entire sample of participants who completed the task.

The sample size was determined by the convenience of students’ willingness to volunteer, but for a within-subjects design, a sample size of 34 provides 80% power to detect a moderate effect size of *d *= 0.5 (Cohen, 1988), and so this was our minimum sample size. We discuss this further in the Discussion section.

RQ1: What effect would visualisations and anecdotes have on medical students’ perception of the efficacy of the polypill?

There were main effects of both anecdote, *F*(2, 111) = 0.5.84, *p *< .004, and icon array, *F*(1111) = 6.79, *p *= .01. Importantly, the two variables also interacted, *F*(2, 1111) = 6.24, *p *= .003. One shows the pattern. When there is no icon array, both the positive anecdote (*M *= 2.55 out of 5; *SD *= 0.86) and the negative (*M *= 2.68 out of 5; *SD *= 0.83) elicited lower efficacy ratings than the no anecdote condition (*M *= 3.09 out of 5; *SD *= 0.69). On the other hand, when there was an icon array, the positive (*M *= 2.91 out of 5; *SD *= 0.68), negative, (*M *= 3.05 out of 5; *SD *= 0.83) and no anecdote (*M *= 2.95 out of 5; *SD *= 0.85) conditions were approximately equivalent. We note that even though numerically, the mean scores all seem close(they range from 2.55 to 3.09), the effect sizes are not trivial. In standardised units, the interaction effects are both greater than 0.6, making them “moderate” in size, per Cohen's well-known effect size benchmarks.^30^ Refer to Tables 1 and 2 for parameter estimates for the mixed-effects models in Figure 1.

RQ2: What effect would visualisations and anecdotes have on medical students’ willingness to recommend the polypill?

**Table 1.** Statistical modelling of student ratings of polypill efficacy: these are the results of the analyses for question 1 – which asked medical students to rate the efficacy of the polypill under different conditions. The data were analyzed with a mixed-effects model with fixed effects for icon array and anecdote and their interaction and with random intercepts for each student. The model converged successfully with no issues. *p*-values were calculated using the Satterthwaite approximation for degrees of freedom. This table presents all fixed-effect estimates from the model.

Effect	Parameter estimate	*SE*	*df*	*t*	*P*
(Intercept)	2.877	0.151	24.1	19.01	<.001
Anecdote (positive anecdote vs no anecdote)	−0.278	0.082	110.7	−3.4	<.001
Anecdote (negative anecdote vs no anecdote)	−0.163	0.082	110.3	−1.99	.05
Icon array (yes icon array vs no icon array)	−0.175	0.067	110.8	−2.61	.01
Anecdote (positive anecdote vs no anecdote) × icon array (yes icon array vs no icon array)	−0.495	0.164	110.7	−3.03	.003
Anecdote (negative anecdote vs no anecdote) × icon array (yes icon array vs no icon array)	−0.503	0.164	110.3	−3.07	.003

Model: Q1 – efficacy ∼ 1 + anecdote + icon array + anecdote:icon array + (1 | Sub#).

**Table 2.** Random components for mixed-effects model for question 1 – efficacy judgments.

Groups	Name	*SD*	Variance	ICC
Sub#	(Intercept)	0.749	0.562	0.782
Residual		0.395	0.156	

**Figure 2.**
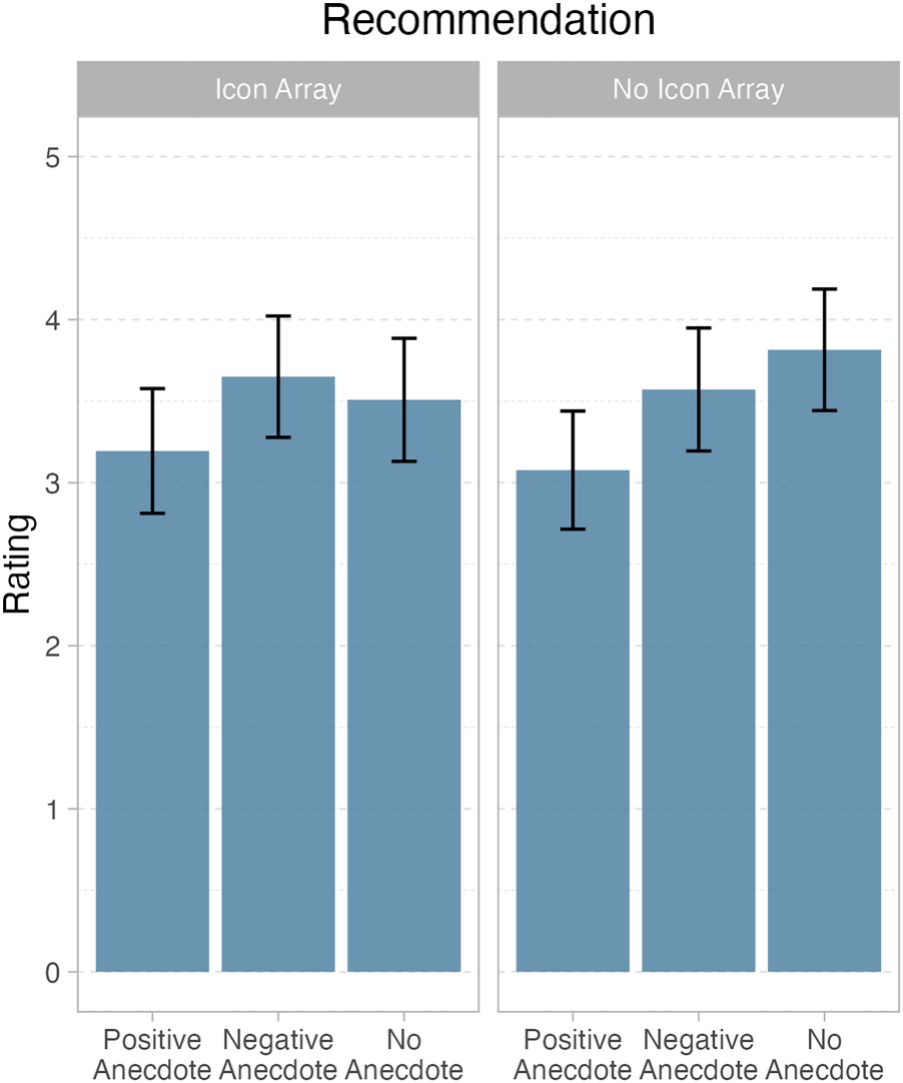
Marginal means and 95% confidence intervals for question 2 – recommendation judgements, showing a main effect of anecdote.

For question 2, there was a main effect of anecdote, *F*(2, 112) = 9.25, *p* < .001. The positive anecdote condition (*M* = 3.23; *SD *= 0.93) elicited less intention to recommend the polypill than either the negative anecdote (*M* = 3.68; *SD *= 0.89) or the no anecdote condition (*M* = 3.77; *SD *= 0.93). There was no effect of icon array, *F*(1, 113) = 0.11, *p* = .74, nor an interaction between the two factors, *F*(2, 112) = 1.51, *p* = .23. Refer to Tables 3 and 4 for parameter estimates and marginal means in Figure 2.

RQ3: What effect did medical students think showing patients visualisations and anecdotes about CVD risk in the consult on adherence to treatment with polypill?

**Table 3.** Statistical modelling of student ratings of willingness to recommend the polypill: these are the results of the analyses for question 2 – which asked medical students to rate their willingness to recommend the polypill under different conditions. The data were analyzed with a mixed-effects model with fixed effects for icon array and anecdote and their interaction and with random intercepts for each student. The model converged successfully with no issues. *p*-values were calculated using the Satterthwaite approximation for degrees of freedom. This table presents all fixed-effect estimates from the model.

Effect	Parameter estimate	*SE*	*df*	*t*	*P*
(Intercept)	3.469	0.141	24.6	24.641	<.001
Anecdote (positive anecdote vs no anecdote)	−0.526	0.135	112.1	−3.891	<.001
Anecdote (negative anecdote vs no anecdote)	−0.051	0.135	111.5	−0.375	.708
Icon array (yes icon array vs no icon array)	−0.037	0.111	112.5	−0.332	.74
Anecdote (positive anecdote vs no anecdote) × icon array (yes icon array vs no icon array)	0.424	0.27	112.1	1.569	.119
Anecdote (negative anecdote vs no anecdote) × icon array (yes icon array vs no icon array)	0.385	0.271	111.5	1.423	.158

Model: Q2 – recommendation ∼ 1 + anecdote + icon array + anecdote:icon array + (1 | Sub#)

**Table 4.** Random components for a mixed-effects model for question 2 – recommendation.

Groups	Name	SD	Variance	ICC
Sub#	(Intercept)	0.653	0.427	0.499
Residual		0.654	0.428	

**Figure 3.**
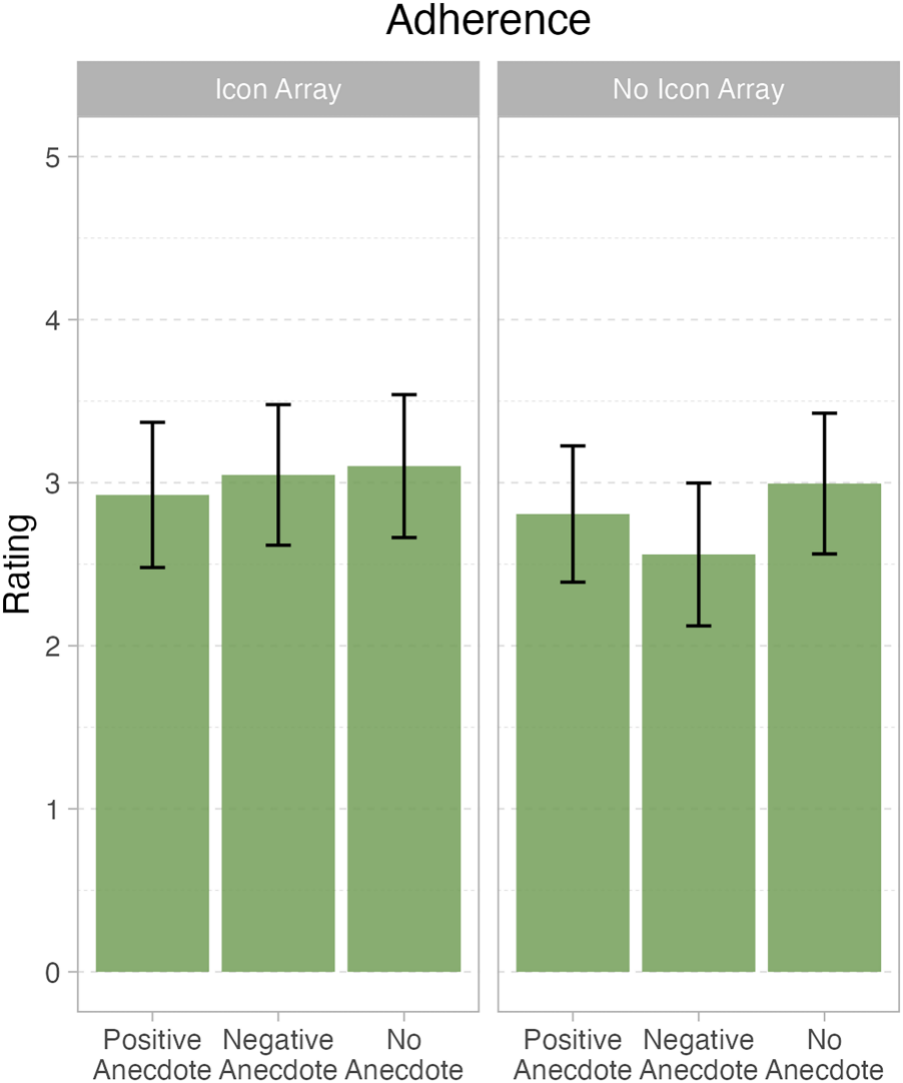
Marginal means and 95% confidence intervals for question 3 – adherence.

This analysis elicited no significantly significant effects, all *F*s < 1.8, all *p*s > .2. Refer to Tables 5 and 6 for parameter estimates and marginal means in Figure 3.

RQ4: What other information do medical students have when engaging in the clinical reasoning process to consider prescribing the polypill?

**Table 5.** Statistical modelling of student ratings of what would aid adherence to taking the polypill: these are the results of the analyses for question 3 – which asked medical students to rate whether the information they were shown would help patients adhere to a prescription of the polypill under different conditions. The data were analyzed with a mixed-effects model with fixed effects for icon array and anecdote and their interaction and with random intercepts for each student. The model converged successfully with no issues. *p*-values were calculated using the Satterthwaite approximation for degrees of freedom. This table presents all fixed-effect estimates from the model.

Effect	Parameter Estimate	*SE*	*df*	*t*	*p*
(Intercept)	2.906	0.152	23.1	19.087	<.001
Anecdote (positive anecdote vs no anecdote)	−0.182	0.170	110.9	−1.067	.289
Anecdote (negative anecdote vs no anecdote)	−0.244	0.171	110.3	−1.431	.155
Icon array (yes icon array vs no icon array)	0.237	0.140	111.6	1.697	.092
Anecdote (positive anecdote vs no anecdote) × icon array (yes icon array vs no icon array)	0.010	0.341	110.9	0.029	.977
Anecdote (negative anecdote vs no anecdote) × icon array (yes icon array vs no icon array)	0.381	0.342	110.3	1.115	.268

Model: Q3 – adherence ∼ 1 + Anecdote + icon array + Anecdote:icon array + (1 | Sub#).

**Table 6.** Random components for a mixed-effects model for question 3 – adherence.

Groups	Name	*SD*	Variance	ICC
Sub#	(Intercept)	0.681	0.464	0.405
Residual		0.826	0.682	

Free texts given in response to the prompt “Are there any other factors you might consider in making the decision to prescribe polypill?” were analysed by one researcher (AJ). These responses were provided by 30 participants, three of whom were undergraduate students and 27 of whom were postgraduate. In total 18 participants provided a free-text response for all six scenarios. The scenarios all had a similar amount of free-text responses, although there was slight variation across all six. Scenario 4 (visual and positive anecdote) generated the most responses at 31. Scenario 1 (visual with negative anecdote) generated the least response with 27. Responses could be classified across four broad categories. Analysis of content related to these categories is presented under relevant sub-headings below.

#### Quality of evidence presented about the polypill clinical trial

This category was used to classify comments where participants critiqued the quality of the clinical trial evidence presented in a case or indicated further information about the trial would be required for them to make a decision about prescribing “polypill.” There was a total of 48 comments related to the quality of evidence presented from the fictitious trial of “polypill,” making it the second most common category of information in the free-text responses. The most common type of content related to this category was requests for additional information about missing demographics particularly in culturally and linguistically diverse populations, followed by “polypill” performance against other therapies and/or drug interactions. Uncommon content included requests for additional information related to participant adherence rates in the trial and the risk of side effects for participants in the trial.

#### More information required about the patient

This category was used to classify comments where participants requested more information about the individual patient they were considering prescribing “polypill” to. There was a total of 59 comments with content related to this category. Sub-categories of content in this category included requests for more information about the patients’ goals; their physical capacity; their individual risk profile; and their family history. Content related to individual risk profiles was overwhelmingly the most prevalent across the scenarios, with 36 comments in total. This was followed by comments indicating that patient goals for their treatment would influence decisions about prescribing “polypill.” There was only one comment requesting information about family history, making it the most uncommon content in this category.

#### More information required about “polypill”

This category was used to classify comments where participants requested more information about “polypill” in the context of prescribing it to a patient. There was a total of 43 comments that were categorised as content related to requests for more information about “polypill.” Sub-categories in this category included queries about alternative therapies; the cost of the drug; and the dosing regimen. Content related to the alternative therapies sub-category was the most common by a significant margin, with a total of 24 comments. This was followed by comments about the cost of “polypill,” 15 comments, and finally dosing regimen, with four comments.

#### Influence of case presentation on prescribing behaviour

This category was used to classify comments where a participant was reflecting on the overall case and their views on prescribing “polypill.” This category had the least amount of content, with only 22 comments from participants. Comments in this category were less homogenous than those in other categories. Sub-categories included comments about being unsure whether further information was required in the case to decide on prescribing; how the scenarios presented side effects; and the need to undertake a further review of the information presented in the case before making a decision to prescribe “polydug.”

## Discussion

Statistical analysis of the responses indicated that the icon array may be effective for highlighting how the polypill reduces CVD risk, reducing the impact of anecdotes on efficacy judgments. Without the icon array, both the positive and negative anecdotes made participants less willing to prescribe the polypill. The inclusion of the icon array did not mitigate the effect of the anecdotes on willingness to prescribe polypill, but there were different effects for positive and negative anecdotes paired. When the positive anecdote was paired with the icon array, it elicited lower ratings for willingness to prescribe polypill. In contrast, the negative anecdote paired with the icon array elicited no different ratings from the no anecdote conditions. Neither the presence of icon arrays nor anecdotes changed the ratings concerning whether or not the participants viewed the information as likely to motivate patients to adhere to taking the polypill. The variable influence of the icon array visualisations when combined with the textual anecdotes may be partially explained by theory on the impact of visualisations on learning processes which suggests the design of information visualisation has to be carefully considered to ensure it is appropriate for the task learners are being asked to undertake; otherwise, visualisations may interfere with construction of knowledge.^18^

Findings from this study indicate that anecdotal information does influence clinical decision-making in medical students. This aligns with the broader literature which has shown that feedback from mentors or peers can influence medical student decision-making,^8^ as can feedback from friends and families.^10^ It also builds on research showing anecdotes influence the general population’s health decision-making,^11^ but showing some level of similar effect with medical students. However, data from this study indicate that the influence of anecdotes on medical student decision-making is not static and can be influenced by the inclusion of other forms of feedback such as icon arrays. Analysis of free-text responses in this study suggested that in addition to the core information presented in a case, medical students draw on a breadth of information when making decisions about prescribing. This finding aligns with the literature which has identified information seeking as a key component of clinical decision-making.^1,8^ Our findings suggested patient preferences were a key piece of information that medical students sought out when making decisions about potentially prescribing a new drug.

Finally, findings from this study suggest that icon arrays on their own and paired with anecdotes influence medical student decision-making. This builds on existing literature on how visualisation influences medical student decision-making, which has shown that visualisation can improve information recall^17^ and the ability to understand how to undertake complex problem-solving.^16^ Our findings also complement existing research suggesting icon arrays are a promising mechanism for communicating medical risk to different patient groups,^31,32^ by showing they may also be effective for communicating similar risks to medical students. The broader literature has shown that visualisations such as icon arrays may also be effective for mitigating different types of cognitive biases when engaging in the decision-making process.^15^ Findings from the study presented in this manuscript suggest icon arrays can be paired with different types of information, such as anecdotes, to influence medical student decision-making. However, study findings are not sufficient to conclude whether this would influence cognitive bias one way or the other.

### Limitations and further research

A limitation of the study is the recruitment of a diverse range of medical students across all years and degree types, which may have created variability in the level of clinical reasoning across the cohort. Further, recruitment was conducted during the COVID-19 pandemic, which is not a typical context under which medical education is delivered and it may have influenced the study cohort.

Another limitation is that our sample size was determined by convenience rather than an a priori power analysis. As we noted above, we set a minimum sample size to be able to resolve moderate effects. Now that this study has established a basis for effect size estimates within a medical student population, future research in this area can calculate power and sample sizes based on these results.

Future researchers should consider further investigating how visualisations and anecdotal information influence medical students’ clinical decision-making, particularly the effect of positive anecdotes. There would also be value in exploring the influence of visualisations and anecdotal information on the decision-making processes of medical professionals, as there is no literature on how these objects influence their decision-making.

## Conclusion

Anecdotal information and visualisations appear to influence the clinical decision-making process of medical students, but the influence of these items appears to be different to that of the general population. Both positive and negative anecdotes about the side effects of a drug appear to make medical students less willing to prescribe it. The inclusion of visualisations such as icon arrays changed the influence of anecdotes on medical student prescribing behaviours, with positive anecdotes about drug side effects paired with visualisations making medical students less willing to prescribe them. It may be beneficial to actively educate medical students about the potential influence of these visualisations and anecdotes on clinical decision-making, particularly given they will be practising medicine in a world where data visualisation and social media anecdotes may be increasingly prevalent in their practice.

## Supplemental Material

sj-pdf-1-mde-10.1177_23821205241293491 - Supplemental material for The Role of Information Visualisation and Anecdotal Evidence in Medical Students’ Clinical Reasoning Process: A Cross-Sectional Survey Study Supplemental material, sj-pdf-1-mde-10.1177_23821205241293491 for The Role of Information Visualisation and Anecdotal Evidence in Medical Students’ Clinical Reasoning Process: A Cross-Sectional Survey Study by Anna Janssen, Micah B. Goldwater, Courtney B. Hilton, Carissa Bonner and Tim Shaw in Journal of Medical Education and Curricular Development

sj-pdf-2-mde-10.1177_23821205241293491 - Supplemental material for The Role of Information Visualisation and Anecdotal Evidence in Medical Students’ Clinical Reasoning Process: A Cross-Sectional Survey Study Supplemental material, sj-pdf-2-mde-10.1177_23821205241293491 for The Role of Information Visualisation and Anecdotal Evidence in Medical Students’ Clinical Reasoning Process: A Cross-Sectional Survey Study by Anna Janssen, Micah B. Goldwater, Courtney B. Hilton, Carissa Bonner and Tim Shaw in Journal of Medical Education and Curricular Development
