# Supplementary material for: The Role of Information Visualisation and Anecdotal Evidence in Medical Students’ Clinical Reasoning Process: A Cross-Sectional Survey Study
Source: J Med Educ Curric Dev. 2024 Nov 21;11:23821205241293491. doi: 10.1177/23821205241293491 (PMC11580097; doi:10.1177/23821205241293491)
Supplement: sj-pdf-1-mde-10.1177_23821205241293491 - Supplemental material for The Role of Information Visualisation and Anecdotal Evidence in Medical Students’ Clinical Reasoning Process: A Cross-Sectional Survey Study [file sj-pdf-1-mde-10.1177_23821205241293491.pdf]

Scenario 1

A large sample placebo-controlled RCT of a new polypill, Polypill D, was recently published. The aim was to see if it reduced the risk of a CVD event in high risk patients. Because high risk is defined as more than 15% risk of CVD event over the next 5 years, the RCT examined people with between a 15% and 30% global CVD risk for a total of 5 years. All participants were non-smokers, did not have diabetes, had no prior history of CVD or chronic kidney disease, and were between the ages of 40 and 65.

The polypill is known to have a number of potential side effects, the main three are hypotension, development of diabetes and experiencing a gastrointestinal bleed. It was reported that 1.7% of participants that received the polypill during the RCT experienced hypo
tension (abnormally low blood pressure), 2.8% of participants who received the polypill during the RCT developed diabetes and 4.4% of participants who received the polypill during the RCT experienced gastrointestinal bleeding. In comparison participants who were in the control arm of the RCT and did not receive the polypill experienced side effects at the following rates:2.3% had blood pressure that was too low (hypotension) 2.4% developed diabetes, and 3.2% experienced gastrointestinal bleeding.

CONTROL GROUP

Of the people in the RCT who were given a place-bo, 22% suffered a CVD event over the course of 5 years.

Grey represents the 78% who do not suffer a CVD event without taking the new polypill.

Red are the 22% who suffered a CVD event in the placebo condition

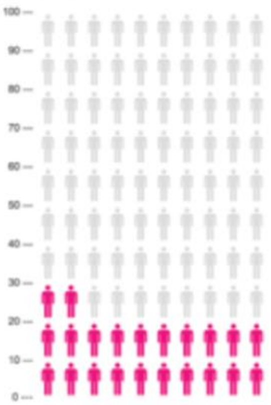

INTERVENTION GROUP

Of the people in the RCT who were given the new polypill 17% suffered a CVD even over the course of 5 years. That is a clinically significant risk reduction of 5%.

Red and Blue/Green add up to the 22% who would suffer a heart attack without the polypill

Blue/Green are the 5% who do not suffer a heart attack, but may have otherwise if they did not take the polypill.

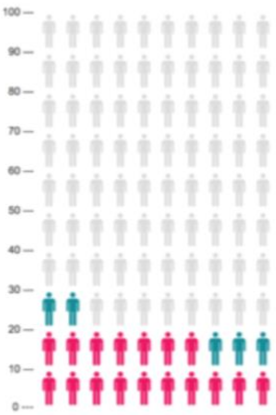

[Anecdote] Further information:

In addition, here’s a case report about an individual from the study.

Roy was a male patient aged 53 with 163/81 mm Hg blood pressure, total serum cholesterol of 6.7 mmol/L, HDL cholesterol* of 1.3 mmol/L, and no history of diabetes, smoking or family CVD. He was not overweight and engaged in adequate exercise.

When Roy entered the RCT these qualities resulted in a 15% risk of having a heart attack or stroke within 5 years, which was considered a high risk. He received the polypill and had not experienced a CVD event as of year 5 of the study. He has also not reported any of the known side effects associated with polypill.

**HDL cholesterol is a 'good' type of cholesterol. CVD risk from cholesterol is calculated as total cholesterol/HDL cholesterol.*

Scenario 2
A large sample placebo-controlled RCT of a new polypill, Polypill A, was recently published. The aim was to see if it reduced the risk of a CVD event in high risk patients. Because high risk is defined as more than 15% risk of CVD event over the next 5 years, the RCT examined people with between a 15% and 30% global CVD risk for a total of 5 years. All participants were non-smokers, did not have diabetes, had no prior history of CVD or chronic kidney disease, and were between the ages of 40 and 65.

Of the people in the RCT Control Group who were given placebo 24% suffered a CVD event over the course of the 5 years.

Of the people in the RCT Intervention group who were given the new polypill 20% suffered a CVD event over the course of 5 years. That is a clinically significant risk reduction of 4%.

The polypill is known to have a number of potential side effects, the main three are hypotension, development of diabetes and experiencing a gastrointestinal bleed. It was reported that 1.7% of participants that received the polypill during the RCT experienced hypotension (abnormally low blood pressure), 2.8% of participants who received the polypill during the RCT developed diabetes and 3.2% of participants who received the polypill during the RCT experienced gastrointestinal bleeding. In comparison participants who were in the control arm of the RCT and did not receive the polypill experienced side effects at the following rates: 2.3% had blood pressure that was too low (hypotension), 2.4% developed diabetes, and 3.2% experienced gastrointestinal bleeding.

<u>[Anecdote] Further information:</u>
In addition, here’s case report about an individual from the study:

Karl was a male patient aged 59 with 155/100 mm Hg blood pressure, total serum cholesterol of 6.4 mmol/L, HDL* cholesterol of 1.0 mmol/L, and no history of diabetes, smoking or kidney disease. He had a family history of CVD, reporting his father had a CVD event at 60. He was not overweight and engaged in adequate exercise. When Karl entered the RCT these qualities resulted in a 15% risk of having a heart attack or stroke within 5 years, which was considered a high risk. He received the polypill and had not experienced a CVD event as of year 5 of the study. He has reported experiencing low blood pressure on one occasion during the study, but has not experienced any of the other known side effects associated with polypill.

**HDL cholesterol is a 'good' type of cholesterol. CVD risk from cholesterol is calculated as total cholesterol/HDL cholesterol.*

Scenario 3
A large sample placebo-controlled RCT of a new polypill, Polypill F, was recently published. The aim was to see if it reduced the risk of a CVD event in high risk patients. Because high risk is defined as more than 15% risk of CVD event over the next 5 years, the RCT examined people with between a 15% and 30% global CVD risk for a total of 5 years. All participants were non-smokers, did not have diabetes, had no prior history of CVD or chronic kidney disease, and were between the ages of 40 and 65.

The polypill is known to have a number of potential side effects, the main three are hypotension (abnormally low blood pressure), development of diabetes and experiencing a gastrointestinal bleed. It was reported that 1.7% of participants that received the polypill during the RCT experienced hypotension, 2.8% of participants who received the polypill during the RCT developed diabetes and 3.2% of participants who received the polypill during the RCT experienced gastrointestinal bleeding. In comparison participants who were in the control arm of the RCT and did not receive the polypill experienced side effects at the following rates: 2.3% had blood pressure that was too low (hypotension), 2.4% developed diabetes, and 3.2% experienced gastrointestinal bleeding

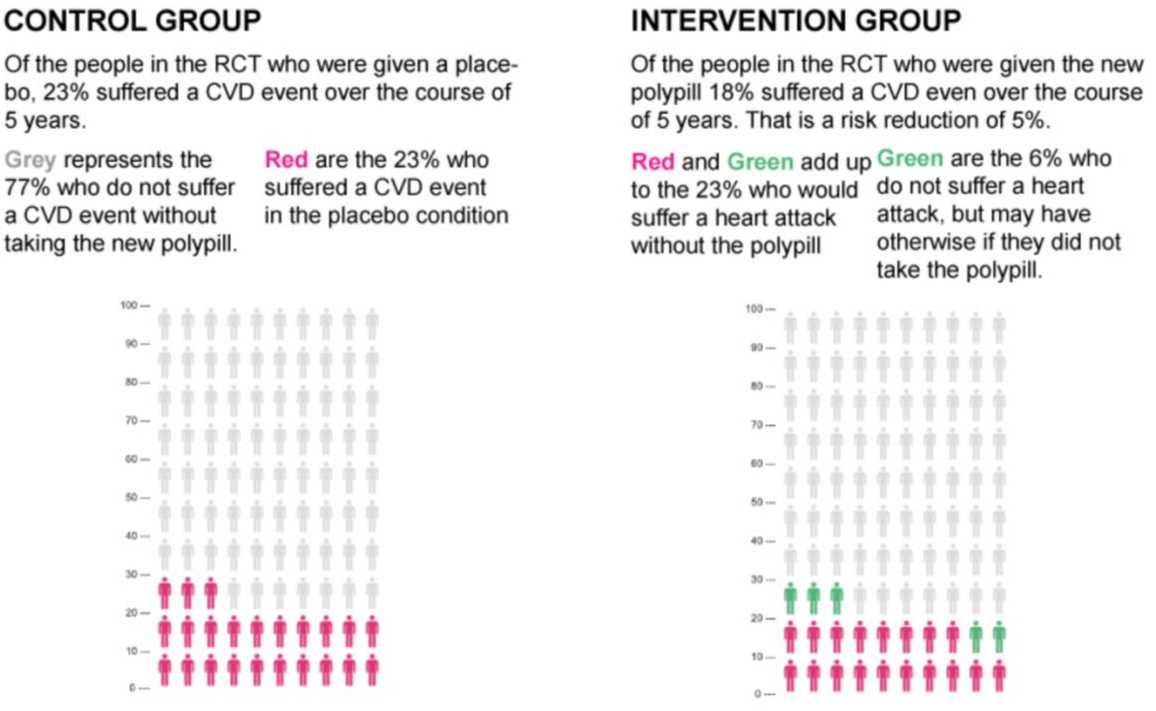

Scenario 4
A large sample placebo-controlled RCT of a new polypill, Polypill E, was recently published. The aim was to see if it reduced the risk of a CVD event in high risk patients. Because high risk is defined as more than 15% risk of CVD event over the next 5 years, the RCT examined people

with between a 15% and 30% global CVD risk for a total of 5 years. All participants were non-smokers, did not have diabetes, had no prior history of CVD or chronic kidney disease, and were between the ages of 40 and 65.

The polypill is known to have a number of potential side effects, the main three are hypotension, development of diabetes and experiencing a gastrointestinal bleed. It was reported that 1.7% of participants that received the polypill during the RCT experienced hypotension (abnormally low blood pressure) 2.8% of participants who received the polypill during the RCT developed diabetes and 3.2% of participants who received the polypill during the RCT experienced gastrointestinal bleeding. In comparison participants who were in the control arm of the RCT and did not receive the polypill experienced side effects at the following rates: 2.3% had blood pressure that was too low (hypotension), 2.4% developed diabetes, and 3.2% experienced gastrointestinal bleeding.

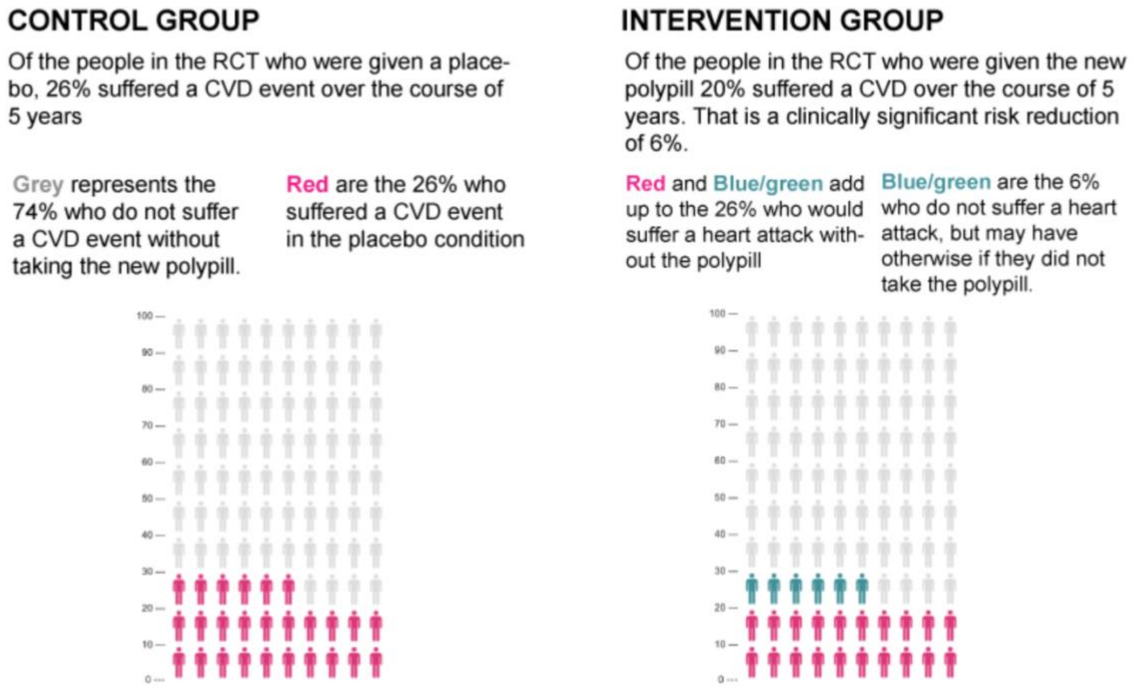

<u>[Anecdote] Further information:</u>
In addition, here's a case report about an individual from the study:

Thomas was a male patient aged 62 with 158/79 mm Hg blood pressure, total serum cholesterol of 6.9 mmol/L, HDL cholesterol* of 1.1 mmol/L, and no history of diabetes, smoking or family CVD. He was not overweight, and engaged in moderate exercise.

These qualities resulted in a 18% CVD risk when Thomas entered the RCT. He received the polypill, and suffered a heart attack in year 3 of the study. By the conclusion of the 5 year study Thomas had also developed diabetes

**HDL cholesterol is a ‘good’ type of cholesterol. CVD risk from cholesterol is calculated as total cholesterol/HDL cholesterol.*

Scenario 5
A large sample placebo-controlled RCT of a new polypill, Polypill C, was recently published. The aim was to see if it reduced the risk of a CVD event in high risk patients. Because high risk is defined as more than 15% risk of CVD event over the next 5 years, the RCT examined people with between a 15% and 30% global CVD risk for a total of 5 years. All participants were non-smokers, did not have diabetes, had no prior history of CVD or chronic kidney disease, and were between the ages of 40 and 65.

Of the people in the RCT Control Group who were given placebo 19% suffered a CVD event over the course of the 5 years.

Of the people in the RCT Intervention group who were given the new polypill 13% suffered a CVD event over the course of 5 years. That is a clinically significant risk reduction of 6%.

The polypill is known to have a number of potential side effects, the main three are hypotension, development of diabetes and experiencing a gastrointestinal bleed. It was reported that 1.7% of participants that received the polypill during the RCT experienced hypotension (abnormally low blood pressure), 2.8% of participants who received the polypill during the RCT developed diabetes and 3.2% of participants who received the polypill during the RCT experienced gastrointestinal bleeding. In comparison participants who were in the control arm of the RCT and did not receive the polypill experienced side effects at the following rates: 2.3% had blood pressure that was too low (hypotension), 2.4% developed diabetes, and 3.2% experienced gastrointestinal bleeding.

Scenario 6
A large sample placebo-controlled RCT of a new polypill, Polypill B, was recently published. The aim was to see if it reduced the risk of a CVD event in high risk patients. Because high risk is defined as more than 15% risk of CVD event over the next 5 years, the RCT examined people with between a 15% and 30% global CVD risk for a total of 5 years. All participants were non-smokers, did not have diabetes, had no prior history of CVD or chronic kidney disease, and were between the ages of 40 and 65.

Of the people in the RCT Control Group who were given placebo 23% suffered a CVD event over the course of the 5 years.

Of the people in the RCT Intervention group who were given the new polypill 19% suffered a CVD event over the course of 5 years. That is a clinically significant risk reduction of 4%.

The polypill is known to have a number of potential side effects, the main three are hypotension, development of diabetes and experiencing a gastrointestinal bleed. It was reported that 1.7% of participants that received the polypill during the RCT experienced hypotension (abnormally low

blood pressure), 2.8% of participants who received the polypill during the RCT developed diabetes and 3.2% of participants who received the polypill during the RCT experienced gastrointestinal bleeding. In comparison participants who were in the control arm of the RCT and did not receive the polypill experienced side effects at the following rates: 2.3% had blood pressure that was too low (hypotension), 2.4% developed diabetes, and 3.2% experienced gastrointestinal bleeding

[Anecdote] Further information:
In addition, here's a case report about an individual from the study:

John was a male patient aged 67 with 157/83 mm Hg blood pressure, total serum cholesterol of 5.3 mmol/L, HDL* cholesterol of 1.2 mmol/L, and no history of diabetes, smoking or kidney disease. He has a family history of CVD, reporting his father had a CVD event at 52. John was not overweight and engaged in adequate exercise. When John entered the RCT these qualities resulted in a 16% risk of having a heart attack or stroke within 5 years, which was considered a moderate risk. He received the polypill and had a CVD event 4 years into the study.

**HDL cholesterol is a 'good' type of cholesterol. CVD risk is from cholesterol is calculated as total cholesterol/HDL cholesterol.*
